# Association Between Anxiety and Suicidal Ideation, and Dietary Patterns

**DOI:** 10.3390/nu18101568

**Published:** 2026-05-14

**Authors:** Mir Jun, Jihyun Woo, Ju-Hye Chung, Se-Hong Kim, Youngmi Eun

**Affiliations:** 1 Department of Family Medicine, Yeouido St. Mary’s Hospital, College of Medicine, The Catholic University of Korea, Seoul 06591, Republic of Korea; mirjack@cmcnu.or.kr (M.J.); wujihyun@gmail.com (J.W.); juana@catholic.ac.kr (J.-H.C.); 2 Department of Family Medicine, St. Vincent’s Hospital, College of Medicine, The Catholic University of Korea, Seoul 06591, Republic of Korea; iron1600@catholic.ac.kr

**Keywords:** anxiety, suicide, macronutrient

## Abstract

**Background/Objectives**: Diet is considered one of the most important modifiable risk factors for non-communicable diseases in modern society. While numerous studies have reported on the association between diet and mental health, including anxiety, research examining the relationship between dietary patterns and mental health is relatively scarce. Therefore, this study aimed to analyze the association between anxiety and suicidal ideation with macronutrient intake. **Methods**: This study was conducted on adults aged 19 years or older using raw data from the 2021–2023 Korea National Health and Nutrition Examination Survey. Excluding those with missing test items, 9002 subjects were included. The study subjects were divided into four groups based on macronutrient intake (normal diet group, high-carbohydrate diet group, high-fat diet group, and high-protein diet group; based on Korean Dietary Reference). **Results**: There was no significant association between dietary patterns and suicidal ideation. However, after adjusting for covariates for moderate or severe anxiety in the HP diet group, the odds ratio was reported to be 0.492 (95% CI 0.298–0.810). Subgroup analysis by gender revealed no significant difference between dietary types and anxiety in women, but in men, the HP diet significantly lowered the odds of moderate or severe anxiety (OR 0.230, 95% CI 0.089–0.599). **Conclusions**: This study found that higher protein intake was associated with lower levels of moderate to severe anxiety, and this trend was statistically significant, particularly in men. Further research is needed to confirm the causal relationship.

## 1. Introduction

The American Psychiatric Association defines mental health as a state of emotional stability associated with appropriate adaptive behavior and relative freedom from anxiety and helplessness. Mental health also encompasses the ability to form constructive interpersonal relationships and effectively cope with everyday demands and stresses [1]. According to a 2019 report, more than 900 million people worldwide suffer from mental illness, and those with serious illness are reported to die an average of 10–20 years earlier than the general population [2].

Mental illness is one factor that is most strongly associated with suicide [3]. According to a 2021 Organization for Economic Co-operation and Development (OECD) report, South Korea’s age-standardized suicide rate per 100,000 people is the highest among all member countries [4]. In 2023, Statistics Korea recorded 13,978 suicide deaths, an 8.3% increase from the previous year. Suicide is the leading cause of death among those aged 10–40 years but, among all suicide deaths, those in their 80s account for the largest proportion, followed by those in their 70s, indicating that suicide is a major cause of death across all age groups [5]. Therefore, managing and promoting mental health, which can influence suicide, is a critical public health challenge [6].

Anxiety disorders are among the most common mental illnesses, with an estimated 4.05% of the global population suffering from such conditions. The prevalence thereof increased by 55% from 1990 to 2019 [7]. In South Korea, the lifetime prevalence of anxiety disorders among adults is higher than that of other mental disorders and has shown a steady increase, rising from 8.7% in 2011 to 9.3% in 2021 [8,9]. Anxiety is defined as a state of worry, concern, or nervousness accompanied by physical, cognitive, and behavioral symptoms [10]. If anxiety is excessive or persistent and interferes with daily life, it can become a pathological condition [11]. Patients with anxiety disorders experience moderate-to-severe psychological distress and functional impairment [12], and high rates of comorbidity with other mental conditions such as major depressive disorder, social phobia, and alcohol dependence [13].

Diet is considered one of the most important modifiable risk factors that influence the non-communicable disease rates of modern society [14]. Nutritional psychiatry is an academic field that investigates, based on evidence, the impact of diet and nutrients on mental and brain health throughout the lifespan and applies these findings to various clinical conditions [15]. Although the mechanisms by which nutritional imbalances cause mental illness have not yet been clearly elucidated, such imbalances may induce chronic inflammation, and inflammatory cytokines have been reported to be closely associated with depression and bipolar disorder [16]. Dietary influences on neurotransmitter systems may also affect stress responsiveness through the serotonin and kynurenine pathways, while dysregulation of the hypothalamic–pituitary–adrenal (HPA) axis may further exacerbate stress-related hormonal responses [17]. Furthermore, alterations in the composition and function of the gut microbiota caused by unhealthy dietary habits can affect central nervous system activity through neuroendocrine and immune signaling pathways [18]. Numerous studies have demonstrated associations between nutrients and mental illness; however, relatively few have examined the relationship between anxiety and macronutrient intake. Therefore, this study aimed to analyze the association between anxiety and suicidal ideation with dietary macronutrient intake patterns.

## 2. Materials and Methods

### 2.1. Subjects

**Figure 1:**
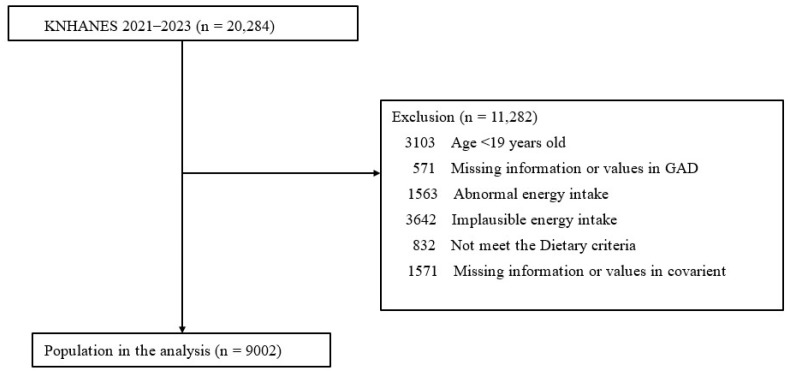
The flow chart of the study.

This study was conducted with adults aged 19 years and older using raw data from the 2021–2023 Korea National Health and Nutrition Examination Survey. Of the 20,284 individuals who participated in the survey, 17,181 were such adults. After excluding those with extreme nutritional intake and those with low nutritional intake reliability, and those for whom data were missing, 9002 individuals remained (Figure 1). This study was approved by the Ethics Committee of the Catholic Medical Center (Institutional Review Board approval number SC25ZISI0047).

Demographic variables retrieved included gender, age, educational level, household income, and employment and marital status. Health behaviors recorded included the body mass index (BMI); smoking, drinking, and physical activity status; and comorbidities (diabetes, hypertension, and dyslipidemia). According to the aerobic physical activity definition of the National Health and Nutrition Survey, such activity was defined as regular if performed for more than 150 min per week at medium intensity, 75 min at high intensity, or a combination thereof. Smoking status was divided into current smokers and non-smokers based on current smoking status. Alcohol consumption was defined as the use of alcohol at least once a month over the past year, based on monthly drinking rates. In terms of comorbidities, diabetes, hypertension, and dyslipidemia status were assessed by reference to medical histories.

### 2.2. Mental Health

This study examined factors related to anxiety, stress, and suicide. Perceived stress was assessed on a scale of 1 to 4, with higher scores indicating greater stress. In terms of suicide, a participant was considered to have a problem if an answer to any of the following questions was “yes”: “Have you ever thought about suicide in the past year?”, “Have you ever planned to commit suicide in the past year?”, or “Have you ever attempted suicide in the past year?”

Anxiety was assessed using the Generalized Anxiety Disorder-7 (GAD-7) screening tool. The GAD-7 was administered via a self-report questionnaire, and trained staff were available to provide explanations when necessary. The GAD-7 questionnaire was developed by Spitzer et al. in 2006 [19]. Each item is scored as follows: 0 = not at all, 1 = several days, 2 = more than half of all days, and 3 = nearly every day. Severity is determined by summing the scores of 7 items (Feeling nervous, anxious, or on edge, Not being able to stop or control worrying, Worrying too much about different things, Trouble relaxing, Being so restless that it is hard to sit still, Becoming easily annoyed or irritable, Feeling afraid as if something awful might happen). The total score ranges from 0 to 21, and anxiety severity is categorized as follows: 0–4, normal; 5–9, mild; 10–14, moderate; and 15–21, severe. The GAD-7 has been reported to have a sensitivity of 81.1% and a specificity of 84.5% [20]. In this study, the cutoff point was set at 10, corresponding to moderate or greater anxiety; scores < 10 were defined as no anxiety whereas scores ≥ 10 were defined as anxiety.

### 2.3. Dietary Assessment

In the Korea National Health and Nutrition Examination Survey, dietary assessment is conducted by registered dietitians or individuals who have majored in nutrition. After selection, investigators complete 2–4 weeks of training and practical instruction before being deployed to the field, and survey performance is continuously evaluated via regular annual training sessions and field quality control. Trained investigators in each region use mobile units to conduct examinations, health interviews, and nutrition surveys. Dietary intake is estimated using a recall method, which includes all foods and beverages consumed by a participant during the previous 24 h. To ensure accuracy, additional tools such as two-dimensional food volume aids, containers, and food models are used. Dietary intake is estimated by reference to the Food Composition Table of the Rural Development Administration and the nutrient database of the Korea Health Industry Development Institute based on the Dietary Reference Intakes for Koreans.

Participants were classified into four dietary groups by the extent of macronutrient intake [a normal group, a high-carbohydrate (HCHO) group, a high-fat (HF) group, and a high-protein (HP) group]. The normal diet group included participants who met the Korean Dietary Reference Intakes for macronutrients (carbohydrates 55–65%, fat 15–30%, and protein 7–20%). An HCHO diet was a diet in which carbohydrates accounted for ≥65% of total energy intake, fat ≤ 30%, and protein ≤ 20%. An HF diet was a diet in which carbohydrates accounted for ≤65%, fat ≥ 30%, and protein ≤ 20%. An HP diet was a diet in which carbohydrates accounted for ≤65%, fat ≤ 30%, and protein ≥ 20%. Energy obtained from carbohydrates, fat, and protein was calculated using standard conversion factors from grams to kilocalories (carbohydrates, 4 kcal/g; fat, 9 kcal/g; protein, 4 kcal/g), and the percentage of total energy contributed by each macronutrient was calculated.

### 2.4. Statistical Analysis

Statistical analyses of raw data followed the guidelines of the Korea National Health and Nutrition Examination Survey, with application of variance estimates and sampling weights based on the complex sampling design. All analyses were conducted using complex sample methods, and statistical significance was defined as a two-sided *p*-value < 0.05. SAS 9.4 software (SAS Institute Inc., Cary, NC, USA) was used for all analyses.

To evaluate differences in general characteristics by the dietary groups, one-way analysis of variance (ANOVA) was used to compare continuous variables, and the chi-squared (χ^2^) test to compare categorical variables. Differences in between-group characteristics by the presence/absence of moderate or severe anxiety (GAD-7 ≥ 10) and suicidal ideation were analyzed using the independent *t*-test for continuous variables and the chi-squared test for categorical variables.

To evaluate associations between dietary patterns and anxiety (GAD-7 ≥ 10) and suicidal ideation, univariate logistic regression analysis was first performed. After adjustment for covariates, complex sample, multivariate logistic regression analysis was then used to calculate odds ratios (ORs) with 95 confidence intervals (CIs). These analyses were performed separately for either gender. Additionally, group comparisons were performed using the chi-squared test to explore differences in suicidal ideation, planning, and attempts by the GAD score.

## 3. Results

### 3.1. Characteristics of the Study Participants

Of the 9002 study participants, the HCHO dietary group was the largest, comprising 4069 individuals (45.2%), whereas the HP group was the smallest, with 769 individuals (8.5%) (Table 1).

When general characteristics were compared by the extent of macronutrient intake, statistically significant differences were observed for all variables, including age, sex, the BMI, marital status, educational level, household income, alcohol consumption, smoking status, physical activity level, total energy intake, dietary fiber and sugar intakes, the prevalence of comorbidities, and the stress level (all *p* < 0.05). The HCHO dietary group exhibited the highest mean age and the greatest dietary fiber and sugar intakes, the largest proportion of married individuals, the greatest prevalence of comorbidities, and the highest levels of stress (levels 3–4). In contrast, the high-fat (HF) dietary group contained the highest proportions of participants with a college education or higher, who were employed, and who enjoyed higher household incomes. The HP dietary group contained the largest proportions of males, those with higher BMI, individuals who consumed alcohol at least once a month, current smokers, and those who engaged in regular aerobic exercise (Table 1).

### 3.2. Mean Values of Covariates by Anxiety and Suicidal Ideation

**Figure 2:**
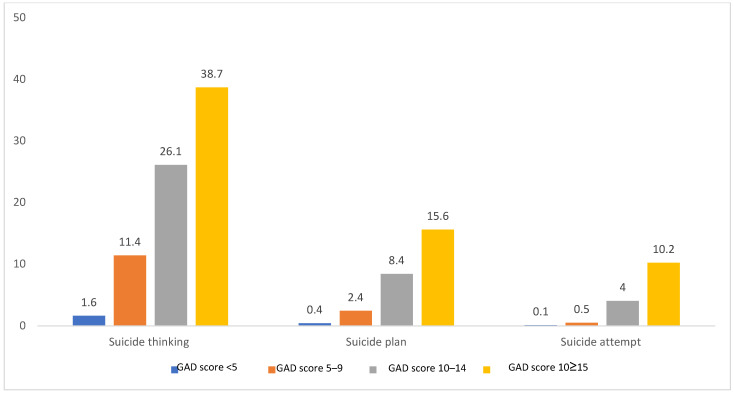
Differences in suicide risk according to anxiety level. There was a statistically significant difference in suicidal thinking, suicidal planning, and suicidal attempt according to the degree of anxiety according to the Generalized Anxiety Disorder (GAD) score (*p* value < 0.001).

Analysis of factors associated with anxiety and suicidal ideation (Table 2) revealed that those with moderate or severe anxiety (GAD-7 ≥ 10) were younger than others, more often female, and were significantly less likely to be married or employed (all *p* < 0.05). In addition, this group evidenced less protein and more carbohydrate intake than others, and tended to suffer less from hypertension. In those with moderate or severe anxiety, the proportions of suicidal ideation, planning, and attempts were significantly higher (all *p* < 0.001). The group exhibiting suicidal ideation contained a higher proportion of unmarried individuals and significantly lower levels of those with graduate-level education and higher household incomes than did other groups (all *p* < 0.05). Significant between-group differences were also observed in terms of smoking status, as well as total energy, dietary fiber, and protein intake. Interestingly, in both the high-anxiety group and those with suicidal ideation, the proportions reporting the highest subjective stress levels (levels 3–4) were lower than those in the control group. When suicidal ideation, planning, and attempts were compared by GAD severity (GAD < 5, 5–9, 10–14, and ≥15) (Figure 2), the proportions of participants rose with increasing GAD scores, and the differences were statistically significant (all *p* < 0.001).

### 3.3. Multivariate Logistic Regression Analysis for the Association Between Dietary Patterns and Anxiety and Suicidal Ideation

Multicollinearity among independent variables was assessed using variance inflation factors (VIFs), and all VIF values were below 5, indicating no significant multicollinearity (Supplementary Table S1). Analysis of the associations between dietary patterns and anxiety and suicidal ideation (Table 3) revealed no significant association. However, in the HP dietary group, the OR for moderate or severe anxiety (GAD-7 ≥ 10) was 0.521 (95% CI, 0.323–0.839) and statistically significant (*p* < 0.05). This association remained significant after adjustment for covariates, including age, sex, socioeconomic factors, health behaviors, and comorbidities; after multivariate adjustment, the OR was 0.492 (95% CI, 0.298–0.810). In subgroup analyses by sex, no significant association between dietary type and anxiety was observed in women whereas, in men, adherence to an HP diet was associated with a significantly lower likelihood of moderate or severe anxiety (OR, 0.230; 95% CI, 0.089–0.599).

## 4. Discussion

In this study, higher protein intake was associated with lower levels of moderate or severe anxiety, and this trend was statistically significant, particularly in men. Protein is essential for maintenance of muscle and skeletal structures, and the homeostasis of other tissues, and may influence not only physical health but also mental stability [21]. Although research on the association between protein intake and anxiety remains limited, several previous studies have reported findings similar to those of the present work. In a study on Indian adolescents, lower intake of protein-rich foods such as milk and soy was significantly associated with a higher prevalence of depression and anxiety, and such symptoms were particularly common among young males who did not consume milk in the morning (depression: OR = 0.28, 95% CI = 0.10–0.77, *p* = 0.0177; anxiety: OR = 0.28, 95% CI = 0.10–0.77, *p* = 0.0177) [22]. In a study of non-diabetic adults in France, protein intake by males exhibited a negative association with the anxiety level (β = −0.01, *p* = 0.05) whereas, in females, carbohydrate intake (total and complex) was positively associated with anxiety and fat intake negatively associated with anxiety (total carbohydrates: β = 0.04, *p* < 0.04; complex carbohydrates: β = 0.05, *p* < 0.02; fat: β = −0.01, *p* < 0.05) [23]. However, a study conducted in Iran reported that higher animal protein intake being significantly associated with greater levels of depression, anxiety, and stress in females [24].

There is ongoing debate regarding the health outcomes of high-protein diets. Levine M. E. et al. analyzed adults aged 50 years and older from NHANES III over an 18-year follow-up period and reported that high protein intake was associated with an increased risk of diabetes-related mortality [25]. In subgroup analyses, individuals aged 50–65 in the high-protein group exhibited increased risks of all-cause and cancer mortality, whereas those aged 65 years and older showed reduced risks of both outcomes, potentially related to age-dependent variations in circulating insulin-like growth factor 1 levels [25]. In addition, findings from the OmniHeart Trial indicated that a high-protein diet (comprising more than 25% of total energy intake) significantly increased estimated glomerular filtration rate, suggesting a potential risk for renal dysfunction [26]. Conversely, a meta-analysis of approximately 30 studies found that higher protein intake was associated with reduced all-cause mortality. Notably, increased consumption of plant-based protein was significantly associated with lower risks of all-cause and cardiovascular mortality, although no significant association with cancer mortality was observed [27]. Furthermore, the evidence regarding the associations between protein intake and the risks of bone disease, kidney disease, and sarcopenia remains inconclusive [28]. The heterogeneity of these findings may be attributable to differences in protein sources, levels of processing, and hormonal variations. Therefore, further well-designed studies are warranted to clarify these relationships.

Amino acids, the basic components of proteins, play essential roles in terms of maintenance of cell function and integrity, including neurons, and high-protein diets have been reported to reduce the risk of mild cognitive impairment and Alzheimer’s disease [29]. In aged mice, long-term low-protein intake induced memory impairment and anxiety-like behavior [30]. Amino acids including tryptophan, tyrosine, and phenylalanine serve as major precursors of neurotransmitters involved in anxiety regulation [24]. In particular, tryptophan is a principal precursor of serotonin and is known to influence serotonergic receptor activity in the brain and regulate cortisol secretion under stress [31]. In a Canadian pilot study on seven participants who ingested defatted pumpkin seeds, which are rich in tryptophan, a significant improvement in anxiety was apparent compared to a control group [32]. Various studies suggest that protein intake could be associated with the physiological mechanisms that regulate anxiety levels, but further research is needed.

An interesting finding of the present study is that, although no direct association was observed between diet and suicidal ideation, higher anxiety levels were significantly associated with increased rates of suicidal ideation, planning, and attempts. Consistent with this finding, a study involving 4882 Chinese medical students also found that higher anxiety levels were significantly associated with greater risks of suicidal ideation and attempts, with a dose–response relationship apparent in terms of anxiety severity [33]. In addition, anxiety comorbid with depression has been reported to further increase the suicide risk [33]. However, a meta-analysis reported that anxiety disorders show only a weak association with suicidal ideation and are not consistently associated with suicidal behavior, with differences depending on the type of anxiety disorder [34]. Suicide is a multifactorial outcome influenced by a range of biological, psychological, and environmental factors, including impulsivity, aggression, hopelessness, and comorbid substance use disorders [35]. Although an association between macronutrient intake and anxiety was observed in this study, no direct association with suicidal ideation was identified. This may suggest that the relationship between dietary factors and suicidality is not direct but rather operates through more complex and potentially indirect pathways involving intermediate affective states and other contextual factors. These findings highlight the need for further longitudinal studies to better elucidate the temporal and mechanistic relationships between diet, mental health, and suicidality.

Another interesting finding was that the proportions of participants reporting higher stress levels (levels 3–4) were in fact lower in the groups with moderate or severe anxiety and/or suicidal ideation. In general, stress is recognized as a major risk factor for mental disorders [36]. However, some studies reported no significant difference in cortisol levels between suicide-attempters and non-attempters, and lower baseline glucocorticoid levels have been associated with reduced reactivity to stress stimuli [37]. These findings may reflect a heterogeneity of the stress-response system, or physiological desensitization; further studies are needed.

This study had several limitations. First, given the cross-sectional design, it was difficult to establish any clear causal relationship. Second, anxiety was assessed using the GAD, a screening instrument, rather than via DSM-5-based diagnostic evaluation. In addition, effects of other psychiatric disorders, such as depression, can not be completely excluded, and the study did not explore the detailed sources of macronutrients or qualitative differences in dietary composition. In particular, extreme energy intake may be associated with underlying psychological or behavioral abnormalities; however, as this is a cross-sectional study, individuals with extreme intake values were excluded to reduce potential bias and to minimize the influence of unmeasured confounding factors. Finally, a limitation is that Korea’s own standards were used instead of the internationally accepted WHO guidelines, which stipulate that carbohydrates account for 45–75% of total daily energy intake for adults, fats less than 30%, and proteins 10–15% [38]. Nevertheless, this study is one of the few large-scale national works to analyze the association between macronutrient composition and anxiety and suicide-related factors, and is significant in that it demonstrates that protein intake was associated with lower anxiety levels, particularly in men.

## 5. Conclusions

Diet and consumption patterns can influence various health behaviors and overall health. In this study, higher protein intake was associated with lower anxiety levels, with a more pronounced association observed in males compared with females. However, no significant association was found between macronutrient intake and suicidal ideation. These findings suggest that while dietary factors may be related to anxiety, their relationship with suicidality is not direct. Given the observed association between higher anxiety levels and increased suicide risk, caution may still be warranted when interpreting the potential mental health implications of dietary patterns. Further studies are needed to clarify the causal relationship between macronutrient intake and anxiety and to elucidate the underlying neurobiological mechanisms.

## Figures and Tables

**Table 1:** General characteristics of the Study Participants.

	Normal Diet	HCHO Diet	HF Diet	HP Diet	*p* Value
Unweighted *n*	2599	4069	1565	769	
Age (year)	49.7 ± 0.4	56.8 ± 0.4	42.4 ± 0.5	47.6 ± 0.7	<0.0001
Gender, %					<0.0001
Male	52.6 (1.0)	48.8 (0.8)	47.6 (1.4)	58.6 (1.9)	
Female	47.4 (1.0)	51.2 (0.8)	52.4 (1.4)	41.4 (1.9)	
BMI (kg/m^2^)	24.1 ± 0.1	24.2 ± 0.1	23.6 ± 0.1	24.3 ± 0.1	0.0001
Marital Status, %					<0.0001
Single	21.7 (1.0)	14.3 (0.8)	36.4 (1.5)	23.8 (2.0)	
Married	78.3 (1.0)	85.7 (0.8)	63.6 (1.5)	76.2 (2.0)	
Education Status, %					<0.0001
High school graduate or lower	52.8 (1.3)	69.8 (1.0)	40.9 (1.5)	51.5 (2.3)	
College degree or higher	47.2 (1.3)	30.2 (1.0)	59.1 (1.5)	48.5 (2.3)	
Employed state, %	64.6 (1.2)	60.4 (0.9)	68.5 (1.3)	67.5 (2.0)	<0.0001
Household income, %					<0.0001
Low	13.6 (0.8)	22.9 (0.9)	9.0 (0.9)	12.3 (1.4)	
Mid	51.6 (1.3)	53.8 (1.1)	52.4 (1.8)	51.8 (2.3)	
High	34.8 (1.4)	23.3 (1.1)	38.6 (1.8)	36.0 (2.2)	
Alcohol, %					<0.0001
<1 time/mon	46.3 (1.2)	56.2 (1.0)	38.5 (1.5)	34.7 (1.9)	
≥1 time/mon	53.7 (1.2)	43.8 (1.0)	61.5 (1.5)	65.3 (1.9)	
Smoking, %					0.0346
Non, Former	81.9 (1.0)	83.2 (0.7)	84.7 (1.1)	79.3 (1.8)	
Current	18.1 (1.0)	16.8 (0.7)	15.3 (1.1)	20.7 (1.8)	
Regular aerobic exercise ^a^, %	48.8 (1.2)	39.4 (1.0)	53.1 (1.5)	54.0 (2.1)	<0.0001
Energy intake (kcal/day)	1801.2 ± 14.9	1646.0 ± 13.1	2090.5 ± 23.1	1804.8 ± 29.1	<0.0001
Carbohydrate intake (g/day)	264.9 ± 2.1	290.2 ± 2.3	238.8 ± 2.5	225.1 ± 3.8	<0.0001
Fat intake (g/day)	46.9 ± 0.4	27.0 ± 0.3	83.5 ± 1.1	42.1 ± 0.8	<0.0001
Protein intake (g/day)	67.8 ± 0.6	52.6 ± 0.5	76.5 ± 1.0	99.5 ± 1.8	<0.0001
Fiber intake (g/day)	25.9 ± 0.3	27.1 ± 0.3	22.6 ± 0.4	24.2 ± 0.5	<0.0001
glucose intake (g/day)	57.0 ± 0.9	59.2 ± 1.0	59.0 ± 1.0	46.8 ± 1.4	<0.0001
Energy from					
Carbohydrate (%)	60.5 ± 0.1	72.2 ± 0.1	47.8 ± 0.2	53.5 ± 0.3	<0.0001
Fat (%)	23.9 ± 0.1	14.8 ± 0.1	37.0 ± 0.2	22.5 ± 0.2	<0.0001
Protein (%)	15.5 ± 0.1	13.0 ± 0.0	15.2 ± 0.1	24.0 ± 0.2	<0.0001
Comorbidity (%)					
Hypertension	27.6 (1.0)	39.1 (0.9)	18.2 (1.2)	28.9 (1.9)	<0.0001
Diabetes	11.6 (0.7)	17.6 (0.7)	7.6 (0.7)	13.8 (1.3)	<0.0001
Dyslipidemia	27.4 (1.0)	31.3 (0.9)	22.1 (1.2)	27.6 (1.8)	<0.0001
Perceived stress ^b^					0.0174
1	3.9 (0.5)	3.7 (0.4)	3.7 (0.5)	3.9 (0.7)	
2	20.0 (0.9)	18.0 (0.8)	21.4 (1.2)	20.4 (1.6)	
3	59.5 (1.2)	59.2 (1.0)	61.0 (1.4)	59.3 (2.0)	
4	16.7 (0.8)	19.0 (0.7)	13.9 (0.9)	16.3 (1.4)	
GAD score	2.0 ± 0.1	1.9 ± 0.1	2.2 ± 0.1	1.9 ± 0.1	0.1686
Suicide thinking, %	4.0 (0.4)	3.9 (0.3)	3.8 (0.6)	2.9 (0.7)	0.6505
Suicide plan, %	1.1 (0.2)	1.1 (0.2)	0.8 (0.3)	0.8 (0.3)	0.7839
Suicide attempt, %	0.6 (0.2)	0.4 (0.1)	0.1 (0.1)	0.2 (0.2)	0.1351

^a^ High-intensity exercise ≥ 75 min/week, or moderate intensity exercise ≥ 150 min/week, or mix moderate and high intensity physical activity (1 min of high intensity equals 2 min of moderate intensity). ^b^ Likert scale of 1 to 4, with 4 being the worst. Values are means ± standard errors or percentages (standard errors). HCHO, high-carbohydrate; HF, high-fat; HP, high-protein; BMI, Body mass index; GAD score, Generalized Anxiety Disorder score.

**Table 2:** Mean values of covariates by anxiety and suicidal thinking.

	GAD		*p*-Value	Suicide Thinking		*p*-Value
	<10	≥10		No	Yes	
Unweighted *n*	8621	381		8641	361	
Age (year)	51.1 ± 0.3	47.9 ± 1.0	0.0009	51.0 ± 0.3	48.8 ± 1.2	0.0649
Gender, %			<0.0001			0.142
Male	51.2 (0.6)	38.0 (3.0)		50.8 (0.6)	46.1 (3.1)	
Female	48.8 (0.6)	62.0 (3.0)		49.2 (0.6)	53.9 (3.1)	
BMI (kg/m^2^)	24.1 ± 0.1	23.7 ± 0.2	0.1448	24.1 ± 0.1	24.0 ± 0.3	0.8513
Marital Status, %			0.0001			<0.0001
Single	21.4 (0.6)	31.2 (2.9)		21.3 (0.6)	35.1 (3.3)	
Married	78.6 (0.6)	68.8 (2.9)		78.7 (0.6)	64.9 (3.3)	
Education Status, %			0.4198			0.0392
High school graduate or lower	57.4 (0.9)	54.9 (3.0)		57.0 (0.9)	63.4 (3.0)	
College degree or higher	42.6 (0.9)	45.1 (3.0)		43.0 (0.9)	36.6 (3.0)	
Employed state, %	64.2 (0.7)	57.4 (3.0)	0.0222	64.5 (0.7)	49.2 (2.9)	<0.0001
Household income, %			0.0709			<0.0001
Low	16.1 (0.6)	21.2 (2.3)		15.9 (0.6)	28.4 (2.6)	
Mid	52.8 (0.9)	50.0 (3.0)		52.8 (0.9)	49.3 (3.1)	
High	31.1 (1.0)	28.8 (2.9)		31.3 (1.0)	22.3 (2.7)	
Alcohol, %			0.0867			0.0554
1 time/mon	47.5 (0.7)	52.8 (3.1)		47.5 (0.7)	53.5 (3.1)	
>1 time/mon	52.5 (0.7)	47.2 (3.1)		52.5 (0.7)	46.5 (3.1)	
Smoking, %			0.1318			<0.0001
Non, Former	82.9 (0.5)	79.1 (2.7)		83.3 (0.5)	68.9 (3.0)	
Current	17.1 (0.5)	20.9 (2.7)		16.7 (0.5)	31.1 (3.0)	
Regular aerobic exercise ^a^, %	46.5 (0.7)	42.1 (2.8)	0.1297	46.4 (0.7)	44.6 (3.1)	0.5825
Energy intake (kcal/day)	1799.6 ± 10.2	1718.7 ± 40.8	0.0512	1800.9 ± 10.0	1678.8 ± 41.8	0.0036
Carbohydrate intake (g/day)	266.5 ± 1.5	261.2 ± 5.3	0.3265	266.8 ± 1.5	254.9 ± 6.4	0.065
Fat intake (g/day)	45.8 ± 0.5	42.2 ± 1.7	0.0366	45.8 ± 0.5	42.2 ± 1.7	0.0379
Protein intake (g/day)	66.5 ± 0.4	61.6 ± 2.3	0.0341	66.5 ± 0.4	60.2 ± 1.8	0.0006
Fiber intake (g/day)	25.6 ± 0.2	24.5 ± 0.7	0.1194	25.7 ± 0.2	24.0 ± 0.8	0.0289
glucose intake (g/day)	57.3 ± 0.6	57.7 ± 2.2	0.8695	57.5 ± 0.6	53.7 ± 2.9	0.1895
Energy from						
Carbohydrate (%)	62.0 ± 0.2	63.9 ± 0.7	0.0046	62.0 ± 0.2	63.2 ± 0.7	0.0823
Fat (%)	22.7 ± 0.1	21.6 ± 0.6	0.0558	22.7 ± 0.1	22.1 ± 0.6	0.3146
Protein (%)	15.3 ± 0.1	14.5 ± 0.2	0.0005	15.3 ± 0.1	14.7 ± 0.2	0.0233
Comorbidity, %						
Hypertension	30.8 (0.7)	24.0 (2.3)	0.005	30.5 (0.7)	31.7 (2.7)	0.6649
Diabetes	13.5 (0.4)	13.1 (1.9)	0.86	13.2 (0.4)	21.1 (2.4)	0.0001
Dyslipidemia	28.0 (0.6)	27.4 (2.4)	0.8171	27.9 (0.6)	28.4 (2.7)	0.8581
Perceived stress ^b^, %			<0.0001			<0.0001
1	2.6 (0.2)	29.6 (2.8)		2.9 (0.2)	24.9 (2.6)	
2	18.1 (0.5)	52.3 (3.0)		18.4 (0.5)	47.7 (2.8)	
3	61.6 (0.6)	15.9 (2.2)		61.1 (0.6)	23.9 (2.8)	
4	17.7 (0.5)	2.3 (0.8)		17.6 (0.5)	3.5 (1.1)	
GAD score	1.5 ± 0.0	13.6 ± 0.2	<0.0001	1.8 ± 0.0	7.4 ± 0.3	<0.0001
Suicide thinking, %	2.7 (0.2)	30.2 (2.6)	<0.0001			
Suicide plan, %	0.6 (0.1)	10.7 (1.8)	<0.0001	-	24.8 (2.5)	-
Suicide attempt, %	0.1 (0.0)	6.0 (1.4)	<0.0001	-	9.1 (1.8)	-

^a^ High-intensity exercise ≥ 75 min/week, or moderate intensity exercise ≥ 150 min/week, or mix moderate and high intensity physical activity (1 min of high intensity equals 2 min of moderate intensity). ^b^ Likert scale of 1 to 4, with 4 being the worst. Values are means ± standard errors or percentages (standard errors). GAD score, Generalized Anxiety Disorder score; BMI, Body mass index.

**Table 3:** Associations between dietary patterns and anxiety and suicidal thinking.

	Unweighted *n*		% (SE)	OR (95% CI)		
	Total *n*	Event *n*		Model 1	Model 2	Model 3
**Outcome: GAD Score ≥ 10**						
**Total**						
**Normal diet**	2599	105	4.17 (0.45)	1 (Ref.)	1 (Ref.)	1 (Ref.)
**HCHO diet**	4069	199	4.88 (0.42)	1.179 (0.887, 1.567)	1.242 (0.915, 1.686)	1.189 (0.871, 1.622)
**HF diet**	1565	53	3.73 (0.55)	0.890 (0.607, 1.306)	0.811 (0.547, 1.200)	0.824 (0.549, 1.235)
**HP diet**	769	24	2.22 (0.49)	0.521 (0.323, 0.839)	0.494 (0.303, 0.807)	0.492 (0.298, 0.810)
**Male**						
**Normal diet**	1205	46	3.95 (0.61)	1 (Ref.)	1 (Ref.)	1 (Ref.)
**HCHO diet**	1780	52	3.22 (0.50)	0.811 (0.533, 1.234)	0.971 (0.618, 1.527)	0.931 (0.593, 1.460)
**HF diet**	650	19	2.93 (0.71)	0.736 (0.410, 1.320)	0.764 (0.414, 1.409)	0.774 (0.412, 1.455)
**HP diet**	395	6	0.89 (0.39)	0.218 (0.087, 0.551)	0.233 (0.091, 0.597)	0.230 (0.089, 0.599)
**Female**						
**Normal diet**	1394	59	4.42 (0.64)	1 (Ref.)	1 (Ref.)	1 (Ref.)
**HCHO diet**	2289	147	6.46 (0.64)	1.494 (1.035, 2.156)	1.500 (1.013, 2.219)	1.434 (0.960, 2.143)
**HF diet**	915	34	4.45 (0.86)	1.008 (0.611, 1.662)	0.878 (0.528, 1.462)	0.896 (0.534, 1.504)
**HP diet**	374	18	4.09 (1.07)	0.924 (0.516, 1.653)	0.768 (0.420, 1.402)	0.767 (0.418, 1.405)
**Outcome: Suicide thinking**						
**Total**						
**Normal diet**	2599	104	4.00 (0.42)	1 (Ref.)	1 (Ref.)	1 (Ref.)
**HCHO diet**	4069	171	3.91 (0.34)	0.975 (0.739, 1.285)	0.979 (0.728, 1.317)	0.832 (0.616, 1.123)
**HF diet**	1565	60	3.83 (0.56)	0.955 (0.666, 1.371)	0.958 (0.663, 1.385)	1.023 (0.695, 1.504)
**HP diet**	769	26	2.90 (0.74)	0.716 (0.415, 1.235)	0.700 (0.396, 1.236)	0.652 (0.362, 1.178)
**Male**						
**Normal diet**	1205	54	4.31 (0.65)	1 (Ref.)	1 (Ref.)	1 (Ref.)
**HCHO diet**	1780	68	3.46 (0.50)	0.794 (0.517, 1.220)	0.879 (0.553, 1.400)	0.742 (0.469, 1.173)
**HF diet**	650	21	2.92 (0.71)	0.667 (0.371, 1.198)	0.705 (0.385, 1.292)	0.734 (0.394, 1.366)
**HP diet**	395	10	2.19 (1.00)	0.497 (0.188, 1.314)	0.517 (0.190, 1.411)	0.489 (0.172, 1.385)
**Female**						
**Normal diet**	1394	50	3.66 (0.58)	1 (Ref.)	1 (Ref.)	1 (Ref.)
**HCHO diet**	2289	103	4.34 (0.46)	1.193 (0.807, 1.764)	1.110 (0.740, 1.664)	0.953 (0.624, 1.457)
**HF diet**	915	39	4.66 (0.86)	1.287 (0.803, 2.063)	1.249 (0.778, 2.005)	1.347 (0.829, 2.189)
**HP diet**	374	16	3.90 (1.10)	1.069 (0.550, 2.078)	0.954 (0.477, 1.908)	0.878 (0.426, 1.813)

Values are expressed as odds ratios (with 95% confidence intervals). GAD score, Generalized Anxiety Disorder score. HCHO, high-carbohydrate; HF, high-fat; HP, high-protein. Model 1: Unadjusted model. Model 2: adjustment for age, sex, smoking, alcohol consumption, aerobic physical activity, and perceived stress. Model 3: Model 2 with adjustment for marital status, household income, daily energy intake, glucose intake, and fiber intake. Results in italics indicate statistical significance at the 0.05 level.

## Data Availability

The data are available at https://knhanes.kdca.go.kr/knhanes/main.do (accessed on 6 April 2026).

## Supplementary Materials

The following supporting information can be downloaded at: https://www.mdpi.com/article/10.3390/nu18101568/s1, Table S1: Variance inflation factors (VIF) for independent variables in Multivariate logistic regression analysis.

## Abbreviations

The following abbreviations are used in this manuscript:

BMI	body mass index
GAD-7	Generalized Anxiety Disorder-7
HCHO	high-carbohydrate
HF	high-fat
HP	high-protein
OR	odds ratio
CI	confidence interval
