# Supplementary material for: Association Between Anxiety and Suicidal Ideation, and Dietary Patterns
Source: Nutrients. 2026 May 14;18(10):1568. doi: 10.3390/nu18101568 (PMC13209512; doi:10.3390/nu18101568)
Supplement: Supplementary file 1 [file nutrients-18-01568-s001.zip › nutrients-4274352-supplementary.pdf]

Supplementary Table S1. Variance inflation factors (VIF) for independent variables in Multivariate logistic regression analysis.

	VIF		
	Total	Male	Female
Diet	1.0398	1.0477	1.0348
Age	2.2395	2.3070	2.2276
Sex	1.4011	-	-
Smoking	1.1853	1.0764	1.0291
Alcohol consumption	1.1981	1.0803	1.1663
Aerobic physical activity	1.0686	1.0732	1.0660
Stress	1.0335	1.0407	1.0279
Household income	1.2476	1.2318	1.2581
Daily energy intake	2.1684	1.7941	2.0566
Fiber intake	2.1327	1.9163	2.2720
Glucose intake	1.7221	1.5827	1.9066
Marital status	1.6704	1.7898	1.5671
